# From Homeostasis to Neuroinflammation: Insights into Cellular and Molecular Interactions and Network Dynamics

**DOI:** 10.3390/cells14010054

**Published:** 2025-01-05

**Authors:** Ludmila Müller, Svetlana Di Benedetto, Viktor Müller

**Affiliations:** Max Planck Institute for Human Development, Lentzeallee 94, 14195 Berlin, Germanyvmueller@mpib-berlin.mpg.de (V.M.)

**Keywords:** neuroinflammation, neuroimmune interactions, microglia, astrocytes, neurons, immune cells, extracellular matrix, multilayer network, network dynamics

## Abstract

Neuroinflammation is a complex and multifaceted process that involves dynamic interactions among various cellular and molecular components. This sophisticated interplay supports both environmental adaptability and system resilience in the central nervous system (CNS) but may be disrupted during neuroinflammation. In this article, we first characterize the key players in neuroimmune interactions, including microglia, astrocytes, neurons, immune cells, and essential signaling molecules such as cytokines, neurotransmitters, extracellular matrix (ECM) components, and neurotrophic factors. Under homeostatic conditions, these elements promote cellular cooperation and stability, whereas in neuroinflammatory states, they drive adaptive responses that may become pathological if dysregulated. We examine how neuroimmune interactions, mediated through these cellular actors and signaling pathways, create complex networks that regulate CNS functionality and respond to injury or inflammation. To further elucidate these dynamics, we provide insights using a multilayer network (MLN) approach, highlighting the interconnected nature of neuroimmune interactions under both inflammatory and homeostatic conditions. This perspective aims to enhance our understanding of neuroimmune communication and the mechanisms underlying shifts from homeostasis to neuroinflammation. Applying an MLN approach offers a more integrative view of CNS resilience and adaptability, helping to clarify inflammatory processes and identify novel intervention points within the layered landscape of neuroinflammatory responses.

## 1. Introduction

Neuroinflammation has emerged as a pivotal component in the pathology of a wide range of neurological disorders, representing a critical response of glial cells to disruptions in nervous tissue homeostasis. Traditionally viewed as a defense mechanism, neuroinflammation is now recognized for its dual nature; while it can be neuroprotective, promote tissue repair, and aid recovery, its chronic or dysregulated form may shift towards neurotoxic states, exacerbating disease progression and contributing to neurological dysfunction [1,2,3].

Two key players are at the heart of neuroinflammation: astrocytes and microglia. These glial cells exhibit a remarkable spectrum of reactivities, which can range from supportive to harmful depending on the context and duration of their activation. In healthy conditions, astrocytes and microglia work synergistically to maintain neuronal health, modulate synaptic activity, and support tissue repair. However, under pathological conditions, their reactivity can become maladaptive, contributing to a vicious cycle of chronic inflammation and neuronal damage [2,4,5].

Neuroinflammation is a multifaceted process involving dynamic interactions between various cellular and molecular components within the CNS. This network encompasses a vast array of interconnected cells, including neurons, glial cells, and immune cells, which communicate through diverse signaling pathways and feedback loops. Recent advances have revealed that the behavior of these cells is profoundly influenced by their microenvironment. The chemical and physical properties of the extracellular matrix play a crucial role in modulating glial cell function and, consequently, neuroinflammatory responses. The dynamic interplay between neural cells and the ECM is further complicated by intense signaling interactions among the various cell types within the nervous system [3,6].

Emerging research has highlighted a new paradigm: the nervous system can be conceptualized as a sophisticated network of cells interconnected through a web of mutual influences and interdependencies. This network perspective emphasizes that intercellular interactions are not merely localized events but are integral components of a broader system where each cell type plays a role in the collective response to both physiological and pathological challenges [1,7].

Interactions between various brain cells and signaling molecules like cytokines, neurotrophins, and neurotransmitters, are complex and dynamic, playing critical roles in modulating brain function, immune responses, and neuroinflammation [2,8,9]. These interactions occur at multiple levels, involving direct signaling pathways, cross-talk between different cell types, and the reciprocal regulation of cytokines as well as the release of neurotransmitters. Understanding the principles of cellular connectivity and interdependence is crucial to unravel the complexity of brain function and dysfunction in health and disease [8,10,11,12].

Intercellular interactions within this network form a web of mutual influences and interdependencies, with each cell type contributing to the overall functioning of the nervous system [1,11]. This interdependent network can be conceptualized as a graph, with cells representing nodes and intercellular interactions representing links or edges. They can either maintain homeostasis or drive maladaptive processes like neuroinflammation. The multilayered nature of this network ensures both robustness under normal conditions and vulnerability in the face of chronic insults or dysregulation. By integrating cellular and molecular data, understanding neuroinflammation through a network approach offers insight into the multi-level organization of inflammatory processes, revealing feedback loops and potential key regulatory hubs, thus paving the way for targeted therapeutic strategies to restore balance and resilience.

In the following sections, we will examine the mechanisms underlying the coordinated interactions between neurons, glial cells, immune cells, and the extracellular matrix, which together integrate diverse signals to generate context-specific and appropriate responses. We will define the main cellular and molecular players within these complex interactions, specifying their phenotypic diversity and functional shifts in response to varying activation states and microenvironmental cues. By exploring these interactions as a multilayered network, we aim to illustrate the complex interplay between cellular and molecular mechanisms that drive neuroinflammatory responses and influence CNS health and pathology.

## 2. The Nervous System as a Dynamic Network: Cellular Interactions and Lifelong Adaptation

The concept of the nervous system as a dynamic system emphasizes its complex organization and continuous adaptation in response to a shifting microenvironment throughout one’s lifespan. Composed of neurons and various supporting cells, such as astrocytes, oligodendrocytes, microglia, and other immune-related cells, the nervous system forms a highly interconnected and responsive network that underlies essential physiological processes, from sensory perception and motor control to cognition and emotional regulation. This responsiveness is not only fundamental for maintaining homeostasis and function but also reflects a remarkable adaptability that allows the nervous system to meet developmental, environmental, and age-related demands [1].

During early development, the nervous system undergoes extensive and precisely coordinated morphological and functional transformations. Neuronal proliferation, migration, and differentiation sculpt neural circuits, creating the foundation for complex neural processes, including perception, motor abilities, cognition, and social behaviors [13]. These developmental processes are tightly regulated by both intrinsic genetic programs and extrinsic environmental factors, with molecular cues such as growth factors, cytokines, and ECM components directing the structural and functional organization of the developing brain [7,14]. For example, specific neurotrophic factors promote axonal growth, synapse formation, and neural differentiation, while glial cells support synaptic refinement by removing unnecessary connections, allowing for a finely tuned neural architecture that can adapt to sensory experiences and environmental inputs.

The microenvironment’s influence on development is profound, with factors like nutrition, early life stress, and toxin exposure shaping the wiring, connectivity, and plasticity of the developing nervous system [13,15,16]. Studies have highlighted how experiences during critical periods can either enhance or compromise neural development, affecting cognitive potential and emotional regulation [15,17]. This plasticity is supported by the ECM, which not only serves as a scaffold for cell attachment and migration but also stores signaling molecules that regulate developmental milestones. Astrocytes and microglia, in particular, dynamically interact with neurons and ECM molecules, modulating synaptic maturation, plasticity, and structural integrity, processes crucial for a resilient and adaptable nervous system [1,6,18].

Neuroplasticity, the nervous system’s capacity to reorganize its structure and functions in response to experiences and environmental demands, underscores its adaptability beyond early development [19,20,21,22]. Throughout one’s life, neural circuits adjust in response to learning, memory, sensory experience, and injury, maintaining functional stability while allowing for behavioral and cognitive flexibility. This plasticity enables the brain to adapt to new challenges, create new memories, and recover from injuries, illustrating the nervous system’s intrinsic capability to remodel its synaptic architecture and functional networks [2,19,20,22,23,24,25].

However, with aging, neuroplasticity diminishes, reducing the nervous system’s capacity to adapt and increasing its susceptibility to age-related neurodegenerative processes. The decline in plasticity is influenced by both intrinsic factors, such as reduced neurogenesis and changes in cellular signaling, and extrinsic factors, including accumulated oxidative stress, inflammation, and metabolic alterations. As the nervous system ages, the ECM’s composition and function also change, affecting cellular adhesion, migration, and signaling pathways, which can impede synaptic plasticity and neuronal health. Furthermore, changes in circulating hormones, neurotransmitters, and immune factors increasingly challenge the maintenance of neuronal and glial functions, contributing to cognitive decline and increased vulnerability to disorders like Alzheimer’s and Parkinson’s diseases [3,13,23,26].

Thus, considering the nervous system as a dynamic system highlights its continuous adaptation across one’s lifespan, integrating genetic predispositions with environmental influences to sustain function and resilience [27]. This perspective underscores how neuronal, glial, and ECM interactions evolve to respond to both developmental cues and age-related challenges, reflecting an intricate balance that maintains CNS homeostasis and adaptability. As this balance shifts with age or under disease pressures, understanding the complex interplay of cellular and molecular mechanisms becomes crucial for developing strategies to promote brain health and mitigate neurodegenerative conditions.

In the following section, we will explore the key cellular players—neurons, glial cells, and immune cells—alongside essential molecular components, including cytokines, neurotrophic factors, extracellular matrix proteins, and neurotransmitters. Each of these elements exhibits distinct functional and morphological states, which drive and respond to the dynamic interplay that supports CNS adaptability, resilience, and, when dysregulated, neuroinflammatory processes.

## 3. The Key Cellular and Molecular Players in Homeostatic and Pathological States

The sophisticated functioning of the nervous system relies on the coordinated actions of multiple cellular and molecular players, each with specific roles in maintaining homeostasis and adapting to changes in the microenvironment, including in neuroinflammatory conditions. Neurons, glial cells, and immune cells are essential mediators of both functional stability and adaptive plasticity, with unique capabilities ranging from neurons transmitting electrochemical signals, which modulate synaptic function and support cellular health in glial cells, to immune cells orchestrating inflammatory responses. Together, these cells form an interconnected network that continuously processes and adapts to internal and external cues [7,11,27,28].

In addition to cellular players, a complex array of molecular components—cytokines, neurotrophic factors, ECM proteins, and neurotransmitters—acts as critical mediators of intercellular communication, guiding cellular responses and modulating interactions. In both homeostatic and neuroinflammatory conditions, these molecules enable precise signaling that influences cellular morphology and function in dynamic ways, allowing the nervous system to adjust to its microenvironment and respond to varying conditions, including the complex demands of neuroinflammation [1,14,29].

### 3.1. Microglia

**Figure 1:**
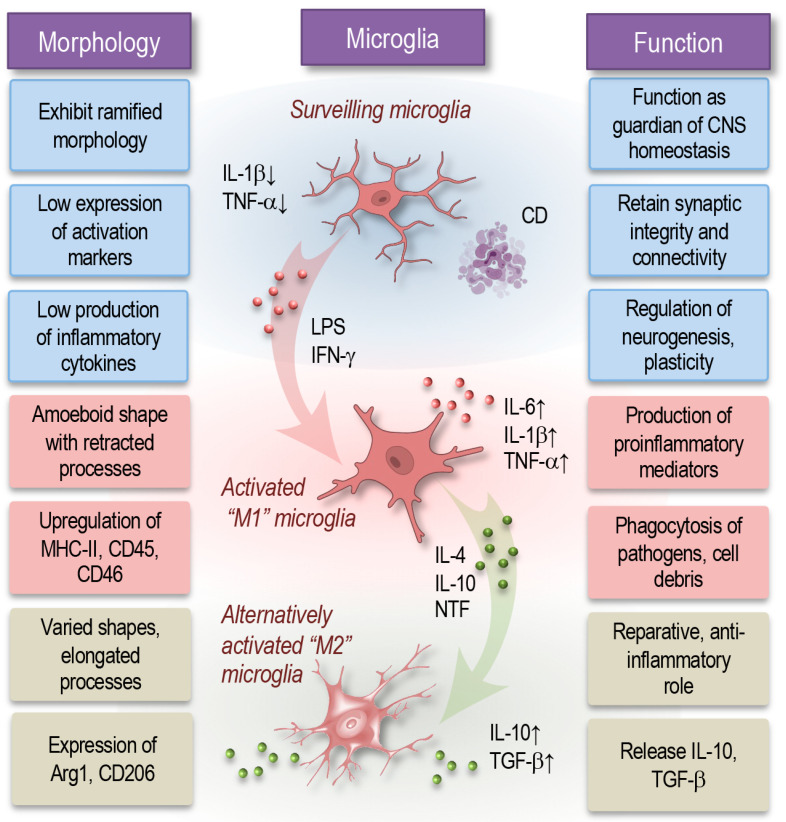
Microglia in the CNS transition between dynamic states in response to environmental cues. In a resting state (blue area), they exhibit a ramified morphology, low activation markers, and perform surveillance, supporting neuronal health, synaptic pruning, and immune defense. Pro-inflammatory stimuli (e.g., LPS and IFN-γ) shift microglia to an “M1” state (red area), with an amoeboid shape, high activation marker expression, and characterized by the production of pro-inflammatory cytokines, driving neuroinflammation. Anti-inflammatory signals (e.g., IL-4, IL-10) promote an “M2” state (green area), associated with tissue repair and anti-inflammatory roles, marked by the expression of Arg1 and CD206. These functional states underscore microglial adaptability in maintaining CNS homeostasis and responding to pathological changes. IL: interleukin; TNF: tumor necrosis factor; ↑: increase; ↓: decrease; CD: cellular debris; IFN: interferon; NTF: neurotrophic factors; TGF: tumor growth factor; CD: cluster of differentiation; MHC: major histocompatibility complex; Arg1: arginase 1.

Microglia, as the resident myeloid cells of the CNS, originate from yolk sac progenitors that migrate into the developing nervous system before the formation of the blood–brain barrier. During these early developmental stages, their migration and positioning are guided by chemokine signaling from neuronal progenitors. Unlike peripheral macrophages, microglia possess a distinct protein profile that reflects their specialized roles in the CNS [1,30]. Under physiological conditions, they are arranged in a well-organized pattern and exhibit a characteristic ramified morphology, with numerous branched processes extending from their cell body (Figure 1). These processes serve as environmental sensors, allowing the microglia to monitor and respond to subtle changes in the CNS [31,32].

In this state, the microglia express low levels of activation markers, including major histocompatibility complex (MHC) class II, cluster differentiation (CD)45, and CD68, along with a subdued production of pro-inflammatory cytokines. This “surveilling phenotype” is functionally adapted to allow for the constant monitoring of the CNS’s microenvironment, enabling microglia to rapidly detect subtle signs of damage or infection. In addition to their surveillance role, resting microglia actively support neuronal health, maintain synaptic integrity through pruning, and provide an immune barrier against pathogens [1,31].

Microglia maintain homeostasis in the nervous tissue through a variety of essential functions, including the regulation of neurogenesis, synaptic density, connectivity, and plasticity. By preventing neurotransmitter toxicity resulting from excessive synaptic release, they play a protective role that is critical for neuronal survival. These functions are modulated by interactions between microglial surface receptors and molecules in the ECM as well as by signals from neighboring cells, highlighting their central position in the CNS network. Furthermore, microglia exhibit cyclic turnover, allowing them to replenish their population and restore proper cell density even after acute cell loss, a process facilitated by cell–cell signaling mechanisms [2,18,33,34].

When exposed to pro-inflammatory stimuli, such as bacterial lipopolysaccharides (LPSs) or cytokines like interferon (IFN)-γ, microglia shift to a classically activated, or so-called “M1” state (Figure 1). This transformation is accompanied by a notable morphological change to an amoeboid shape with retracted processes, reflecting a heightened activation state. M1 microglia exhibit an elevated expression of activation markers, including MHC class II, CD45, and CD86, and secrete pro-inflammatory cytokines such as tumor necrosis factor (TNF)-α, IL-1β, and IL-6 [35,36]. Functionally, M1 microglia play a central role in neuroinflammatory responses, producing pro-inflammatory mediators, enhancing the phagocytosis of pathogens and debris, and promoting neuroinflammation [32,37,38,39].

The transition of microglial function from neuroprotective to neurodegenerative is often time-dependent. When homeostasis is restored, microglia typically revert to a resting state; however, prolonged activation and sustained cytokine release can intensify neuroinflammation, leading to neurodegeneration. In their activated state, microglia perform critical roles, including phagocytosing pathogens, misfolded proteins, cell debris, and apoptotic neurons, which is essential for managing infections and clearing potentially neurotoxic substances [5,30,32].

In response to anti-inflammatory cytokines like IL-4 and IL-10, as well as neurotrophic factors, microglia can also adopt an alternatively activated, or “M2”, phenotype. M2 microglia exhibit varied morphologies, sometimes presenting elongated processes, which are indicative of their reparative and anti-inflammatory roles (Figure 1). These cells are marked by the increased expression of molecules such as arginase-1 (Arg1) and CD206, and they also secrete anti-inflammatory cytokines, including IL-10 and tumor growth factor (TGF)-β. The M2 state facilitates tissue repair and the resolution of inflammation, supports tissue remodeling, and provides neuroprotective effects essential for restoring homeostasis following injury or inflammation [40,41,42].

It is important to note that although we describe microglial activation with three traditionally classified stages, surveilling, pro-inflammatory (M1), and anti-inflammatory (M2), this simplified model does not adequately represent the dynamic and heterogeneous nature of microglial states. Emerging evidence demonstrates that microglial activation exists on a continuum rather than being restricted to discrete states. This continuum reflects the diverse roles of microglia in maintaining homeostasis, responding to injury, and mediating pathological processes [1,43,44,45].

Recent advances in single-cell transcriptomics and proteomics have unveiled novel microglial subpopulations that emerge in response to specific pathological stimuli, offering deeper insights into microglial heterogeneity. For instance, disease-associated microglia (DAM) represent a unique subpopulation identified in neurodegenerative conditions such as Alzheimer’s disease. DAM are characterized by a distinct transcriptional profile regulated by triggering receptors expressed on myeloid cells (TREM)2 signaling and apolipoprotein E (APOE)-dependent pathways. This activation enables them to respond to amyloid-beta plaques, damaged neurons, and other hallmarks of neurodegeneration by adopting roles such as phagocytosis and the secretion of inflammatory mediators. These features position DAM as both protective and potentially deleterious contributors, depending on the context of their activation [38,45,46,47,48,49].

Similarly, lipid-droplet-accumulating microglia (LDAM) have been linked to aging and metabolic disturbances. LDAM are defined by the intracellular buildup of lipid droplets, which impairs their phagocytic efficiency and alters their inflammatory response. These lipid-laden microglia show a heightened production of pro-inflammatory cytokines and oxidative stress markers, contributing to chronic neuroinflammatory conditions observed in aging brains. The presence of LDAM has also been associated with neurodegenerative diseases and metabolic syndromes, suggesting a broader implication in systemic and central nervous system pathologies [45,49].

These findings underscore the flexibility of microglia, which can adopt diverse functional phenotypes in response to specific environmental cues, such as cytokine profiles, tissue damage, and metabolic stress. This plasticity enables microglia to participate as both defenders and facilitators of CNS health, modulating their functional and morphological states in response to shifting microenvironmental cues. Understanding these phenotypic shifts the molecular signals that drive them is critical to unraveling the complex role of microglia in both physiological maintenance and neuroinflammatory responses.

### 3.2. Astrocytes

Astrocytes are a diverse cell population with distinct functional traits influenced by their microenvironment. In the cerebral cortex, astrocytes originate from multiple sources, including radial glia, subventricular zone progenitors, and glial-restricted progenitor cells. In mouse neocortex, astrocytes show various morphological and transcriptional characteristics, where recent studies have identified five astrocyte subtypes with unique markers distributed across brain regions like the olfactory bulb, neocortex, thalamus, and spinal cord [1,50]. This diversity likely results from both environmental factors and interactions with other cell types. Notably, neurons shape astrocyte specialization by releasing signals that help the astrocytes adapt to local neural circuitry needs, highlighting the reciprocal influence between neurons and astrocytes [2,10,51].

**Figure 2:**
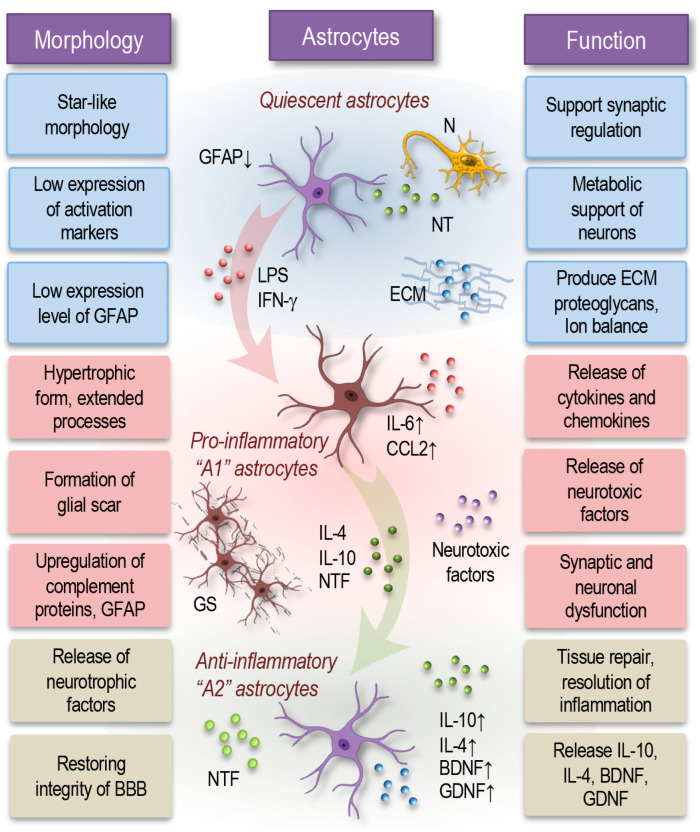
Astrocytes exhibit three distinct phenotypes that play unique roles in CNS homeostasis and neuroinflammation. In a quiescent state (blue area), astrocytes support synaptic function, regulate neurotransmitter levels, and provide metabolic support, releasing molecules like glutamine and D-serine. They also promote synaptogenesis and modulate the ECM to maintain a stable neural environment. During neuroinflammation, astrocytes can transition to either a pro-inflammatory (A1) or an anti-inflammatory (A2) reactive state. A1 astrocytes (red area), activated by signals from microglia or neurons, upregulate complement cascade proteins, release neurotoxic factors, and contribute to neuroinflammation and neuronal damage. Morphologically hypertrophic, they express markers like GFAP and secrete cytokines (e.g., IL-6, CCL2), forming glial scars to surround damaged areas. In contrast, A2 astrocytes (green area), induced by anti-inflammatory signals like IL-4 or IL-10, facilitate tissue repair and inflammation resolution. They express neurotrophic factors (e.g., GDNF and BDNF), anti-inflammatory cytokines, and detoxifying enzymes, promoting neuroprotection and neuronal survival. These astrocytic phenotypes underscore the diverse roles astrocytes play in both protective and pathological responses in the CNS. N: neuron; GFAP: glial fibrillary acidic protein; NT: neurotransmitter; LPS: lipopolysaccharides; IFN: interferon; ECM: extracellular matrix; IL: interleukin; ↑: increase; ↓: decrease; CCL2: CC-chemokine ligand 2; GS: glial scar; NTF: neurotrophic factors; BDNF: brain-derived neurotrophic factor; GDNF: glial cell line-derived neurotrophic factor.

In a healthy CNS, astrocytes maintain a resting or quiescent state with a characteristic star-like morphology and a minimal expression of activation markers (Figure 2). These resting astrocytes play crucial roles in neuronal homeostasis by providing metabolic support, regulating neurotransmitter levels, and contributing to the blood–brain barrier. They exhibit low levels of glial fibrillary acidic protein (GFAP) and produce few cytokines, supporting synaptic regulation, ion balance, and metabolic functions essential for neural stability [52,53].

Astrocytes modulate synaptic function by releasing glutamine (a precursor for neurotransmitters like glutamate and GABA), co-agonists like D-serine, and by regulating neurotransmitter levels in synapses. They also support synaptic plasticity in both developing and mature brains, provide neurons with metabolic support, and influence synaptogenesis via proteins like HEVIN and SPARC [54,55]. Astrocytes can promote neurite regeneration through ECM components like fibronectin, although direct cell contacts can also inhibit outgrowth. Furthermore, astrocytes actively shape the nervous system’s environment by producing ECM proteoglycans and modulating blood flow via COX-1, which aligns blood vessel dilation with neuronal activity. This complex meshwork of astrocytic interactions forms a dynamic support system critical for maintaining neural function and adaptability across the nervous system [1,18,56,57].

During neuroinflammation, some protoplasmic astrocytes proliferate, creating two distinct cell subsets [39,58]. Non-proliferative astrocytes remain in their original locations, maintaining their process domains, while proliferative astrocytes contribute to the formation of a “glial scar”. This scar surrounds and isolates damaged tissue, playing a key role in blocking leukocyte infiltration and restoring the blood–brain barrier [1,59]. Transcriptomic studies in rat models of multiple sclerosis reveal that reactive astrocytes can adopt two opposing phenotypes: a neurotoxic, pro-inflammatory A1 type and a neuroprotective, anti-inflammatory A2 type. However, like the M1/M2 paradigm in microglia, these classifications simplify a complex spectrum of astrocytic responses within the inflamed nervous system [1,51,60]. 

Pro-inflammatory (A1) astrocytes, traditionally identified as a reactive subtype, activate due to signals from microglia or neurons. These A1 astrocytes upregulate complement cascade proteins (e.g., C3 and C4), lose some homeostatic functions, and release neurotoxic factors, promoting neuroinflammation, synaptic dysfunction, and neuronal damage [61]. When responding to injury, inflammation, or neurodegenerative diseases, astrocytes enter a reactive state, marked by morphological changes to a hypertrophic form, with extended processes and an elevated GFAP expression (Figure 2). Reactive astrocytes express additional markers like vimentin and nestin, along with an increased in the production of cytokines and chemokines such as IL-6 and CCL2. These cells actively form glial scars to shield damaged tissue, release neuroprotective factors, and modulate inflammation to support tissue repair [51,58,62,63].

In contrast, anti-inflammatory (A2) astrocytes are induced by anti-inflammatory cytokines such as IL-4 or IL-10 and are linked to tissue repair and inflammation resolution [64]. A2 astrocytes express neurotrophic factors, including glial cell line-derived neurotrophic factor (GDNF) and brain-derived neurotrophic factor (BDNF), alongside anti-inflammatory cytokines and detoxifying enzymes. These astrocytes provide neuroprotection, support neuronal survival, and facilitate tissue repair, working to restore a healthier environment in the CNS [65,66,67,68].

Thus, astrocytes, through their dynamic responses and diverse functional states, play a pivotal role in shaping neuronal health, modulating synaptic activity, and maintaining an optimal environment for neural function and resilience across both healthy and diseased states. In the context of neuroinflammation, astrocytes contribute actively by adopting reactive phenotypes that can either support neuroprotection or exacerbate neuronal injury. They release neurotrophic factors and cytokines, modulate synaptic signaling, and help to maintain the integrity of the blood–brain barrier, all of which can be vital for containing inflammation and minimizing neuronal damage. However, prolonged activation can shift astrocytes toward a more pro-inflammatory state, where they may contribute to neurotoxicity and synaptic dysfunction. Through this dual potential, astrocytes play an essential and complex role in the progression and resolution of neuroinflammatory conditions, profoundly impacting neuronal health and network stability.

### 3.3. Neurons

Neurons are the primary functional units of the nervous system, responsible for transmitting electrical signals and processing information. Through extensive networks formed by synaptic connections, neurons establish pathways where the axons of one neuron connect with dendrites, cell bodies, or the axon terminals of other cells. These synaptic connections represent critical links in the neural network, allowing for signal transmission across excitatory and inhibitory pathways. This architecture enables neurons to carry out complex information processing, integration, and communication within intricate neural circuits, underlying every sensory, motor, and cognitive process [69,70].

During neurogenesis, the formation and integration of new neurons into these circuits depend on a finely tuned interplay with the surrounding glial cells and various molecular signals in the neural microenvironment [71,72]. Astrocytes and microglia, for instance, play crucial roles by releasing growth factors, modulating synaptic plasticity, and influencing synapse formation, all of which are essential for the maturation and integration of new neurons [73]. In healthy conditions, these cellular interactions maintain neural stability and promote adaptability within the system [72]. However, under neuroinflammatory conditions, this balance can be disrupted, with changes in cytokine levels, glial reactivity, and oxidative stress impacting neurogenesis. These inflammatory factors may inhibit neuronal proliferation and survival, thereby impairing circuit integration and overall cognitive resilience. Thus, the collaborative actions of neurons, glia, and molecular players are essential for neurogenesis, impacting both healthy brain function and responses to inflammatory states [74,75].

Neurons are essential to the nervous system, forming complex networks that drive communication, sensation, movement, and cognition [29,76,77]. They exhibit a remarkable adaptability, altering their function and connectivity to respond to internal and external stimuli. This plasticity allows neurons to not only adapt to changing demands and experiences but also to recover, to a degree, from injury. Through intricate synaptic interactions and signaling pathways, neurons coordinate with other neural cells to maintain homeostasis and support brain health [29,55,71].

In neuroinflammatory conditions, however, neurons may suffer from disrupted signaling, oxidative damage, and metabolic stress, making their ability to function optimally highly dependent on the supportive roles of the surrounding cells, especially astrocytes. Astrocytes not only maintain extracellular ion balance and supply metabolic substrates to neurons but also modulate synaptic activity through gliotransmission. These relationships underscore the fundamental interconnectedness of neurons and glial cells in both sustaining neural function and fostering recovery [10,78].

During neuroinflammation, neurons themselves also exhibit significant adaptive changes in response to inflammatory mediators released by reactive glial cells. Neuronal responses to pro-inflammatory signals can lead to altered synaptic function and increased vulnerability to excitotoxicity, further influencing the inflammatory environment. In this context, neurons do not solely act as passive recipients of glial support; their signaling can impact astrocyte and microglial activity, feeding back into the inflammatory cycle. This reciprocal relationship underscores how neuronal and glial crosstalk is essential in both mitigating damage and driving potential recovery within the inflamed nervous tissue [39,58,74,79].

Thus, in addition to their intrinsic electrical signaling properties, neurons operate in tandem with glial cells to maintain homeostasis, adapt to environmental changes, and respond to injury. In healthy conditions, these coordinated interactions preserve neural network stability, allowing for robust sensory processing, memory formation, and adaptation. However, in neuroinflammatory conditions, the interplay between neurons and glial cells becomes disrupted, which can impair synaptic function, alter circuit dynamics, and promote metabolic stress on neurons. The protective role of astrocytes, which can regulate neurotransmitter levels and provide metabolic support, becomes especially crucial for sustaining neural function and supporting recovery in such conditions. This cooperative relationship between neurons and glial cells highlights the interconnected nature of the nervous system, where cellular and molecular synergy is essential for both routine function and resilience in the face of stress or injury.

### 3.4. Immune Cells

**Figure 3:**
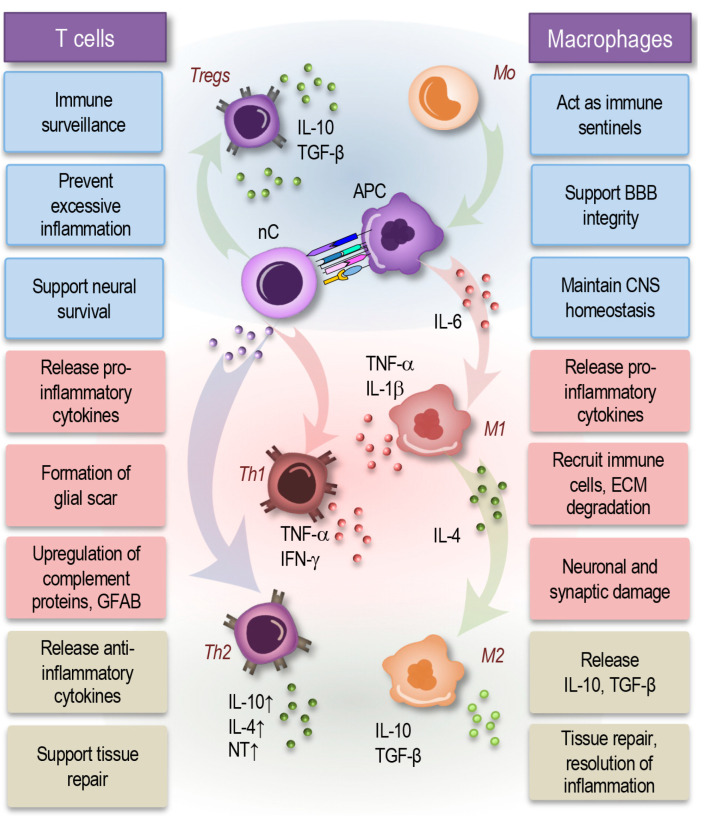
In the CNS, T cells and macrophages perform essential, adaptable roles in both health and disease states. In a healthy CNS, T cells (left) patrol the brain’s borders, including the meninges and choroid plexus, where they maintain immune surveillance and prevent excessive infiltration that could disrupt neural function. Regulatory T cells (Tregs) release anti-inflammatory cytokines like IL-10 and TGF-β, helping to preserve neuronal health by preventing excessive immune activation. Macrophages (right) at CNS borders serve as immune sentinels, clearing antigens and supporting blood–brain barrier integrity. During neuroinflammation, T cells become more prominent within the CNS, with pro-inflammatory Th1 cells releasing cytokines like IFN-γ and TNF, which can exacerbate neuronal stress. Conversely, Th2 cells release IL-4 and IL-10 to help moderate inflammation and promote tissue repair. Macrophages respond by adopting either an inflammatory M1-like phenotype, which releases TNF-α and IL-1β, or a reparative M2-like phenotype, which aids in tissue repair and resolves inflammation. Both T cells and macrophages are also modulated by neurotransmitters like dopamine and serotonin, aligning their immune activity with CNS signaling and ensuring a balance between protection and neuronal preservation. Tregs: regulatory T cells; Th1: T-helper cells 1; Th2: T-helper cells 2; Mo: monocytes; IL: interleukin, ↑: increase; ↓: decrease; nC: naïve cells; APC: antigen-presenting cell; M1: macrophage M1-like phenotype; M2: macrophage M2-like phenotype; IFN: interferon; NT: neurotransmitters; TGF: tumor growth factor.

In the CNS, immune cells, primarily T cells and macrophages, perform essential roles that maintain health, modulate inflammatory responses, and adapt to different physiological conditions (Figure 3). These immune cells support CNS homeostasis in healthy states, while in inflammatory conditions, they engage in complex interactions that influence both protective and potentially harmful pathways [80,81].

T cells in a healthy CNS, although limited in number, provide a supportive role by patrolling the brain’s borders, including the meninges, choroid plexus, and perivascular spaces. Here, they detect potential threats while minimizing immune cell infiltration that could disrupt neural function (Figure 3, left). In this steady state, regulatory T cells (Tregs) help prevent excessive immune activation, which is particularly important given the CNS’s vulnerability to inflammation. Tregs release anti-inflammatory cytokines, such as IL-10 and TGF-β, to counteract minor inflammatory cues and protect neuronal integrity [80,81].

T cells, particularly neuroprotective CD4+ T cells, play a role in regulating neuronal health by directly interacting with neurons and releasing neurotrophic factors or cytokines that support neuronal survival. During inflammatory responses, however, certain T cell subtypes, like Th1 cells, secrete IFN-γ, which can contribute to synaptic dysfunction and neuronal stress [11,80,82,83].

In response to neuroinflammation, such as in autoimmune diseases or neurodegenerative conditions, T cells become more prominent within the CNS. Antigen-presenting cells (APCs) activate T cells by presenting CNS-derived antigens, triggering their differentiation into various T-helper (Th) subtypes. Th1 cells release pro-inflammatory cytokines like IFN-γ and TNF, fostering a response intended to combat pathogens but may also exacerbate neuronal stress if left unchecked (Figure 3, left). Conversely, Th2 cells secrete anti-inflammatory cytokines, such as IL-4 and IL-10, to moderate inflammation and potentially support tissue repair [80,81,84,85,86].

Immune cells and microglia are closely connected through a bidirectional communication system. T cells, for instance, secrete cytokines like IFN-γ and TNF that activate microglia, shifting them to pro-inflammatory states (M1-like). Activated microglia then release signaling molecules, including IL-1β, IL-6, and chemokines, which recruit more T cells and macrophages to the CNS. Conversely, T cells such as Tregs produce anti-inflammatory cytokines like IL-10 and TGF-β, which help the microglia revert to a homeostatic (M2-like) state [5,8,28,38,40].

Astrocytes respond to immune cell signaling by modulating their own activity and releasing cytokines or growth factors that can either amplify or resolve inflammation. During neuroinflammation, activated T cells and macrophages promote astrocyte reactivity, triggering them to release cytokines (e.g., IL-6) that further attract immune cells and amplify the response. In contrast, astrocytes can release anti-inflammatory molecules to signal to immune cells to reduce their activity, fostering tissue repair and dampening neuroinflammation [10,39,51].

Macrophages derived from bone marrow populate the CNS’s borders, including the perivascular spaces, choroid plexus, and meninges (Figure 3, right). In healthy conditions, these cells act as immune sentinels that surveil the CNS for potential pathogens [87]. They serve critical roles in maintaining CNS homeostasis by facilitating antigen clearance and supporting the BBB’s integrity. This state allows macrophages to provide local defense without risking neurotoxic inflammatory reactions [87,88,89]. Depending on the surrounding cytokine environment, macrophages can adopt diverse activation states. For example, IL-4 exposure promotes an anti-inflammatory M2-like phenotype, which enhances tissue repair and supports BBB function [87].

In inflammatory conditions, macrophages quickly respond to cytokines in the CNS’s microenvironment, differentiating into either an inflammatory M1-like phenotype, driven by IL-6 and other pro-inflammatory cytokines, or a reparative M2-like phenotype, promoted by IL-4. M1-like macrophages release inflammatory mediators, such as TNF-α and IL-1β, to activate and recruit other immune cells, helping to fight infection or clear debris from injury sites (Figure 3, right).

However, prolonged activation of M1 macrophages can lead to collateral damage, contributing to chronic inflammation and potentially worsening neuronal damage. In contrast, M2 macrophages play roles in tissue repair by resolving inflammation, clearing apoptotic cells, and remodeling extracellular matrix components, which can help restore CNS function post-injury [37,88].

Both T cells and macrophages in the CNS are not only responsive to cytokines but can also be influenced by neurotransmitters such as acetylcholine, dopamine, and serotonin [83,90,91,92]. These neurotransmitters, which are integral to learning and memory processes, affect immune cells by modulating their production of inflammatory factors. For instance, dopamine and acetylcholine tend to stimulate immune responses, while serotonin and epinephrine exhibit immunosuppressive effects [83,93]. Such neurotransmitter interactions enable immune cells to modulate their activity in synchrony with neuronal signals, ensuring balanced immune responses that align with CNS functional states.

In neuroinflammatory conditions, macrophages and T cells produce enzymes, such as matrix metalloproteinases (MMPs), that degrade ECM components. This degradation is crucial during inflammation as it allows immune cells to migrate through the ECM to reach sites of injury or infection. However, excessive ECM degradation can compromise CNS integrity and exacerbate neuroinflammation [94,95]. Following injury, macrophages work with reactive astrocytes to form a glial scar. The scar is composed of ECM molecules like collagen and proteoglycans, which act as a barrier to inflammation and protect the surrounding neurons. While this is beneficial in the short term, prolonged scar formation can prevent axonal regrowth and repair, illustrating the complex balance immune cells maintain in CNS remodeling [6,18,96,97].

Thus, the roles of T cells and macrophages in the CNS demonstrate a remarkable adaptability to both healthy and inflammatory states. In healthy conditions, these cells maintain a quiet, regulatory presence to prevent unnecessary inflammation. In neuroinflammatory conditions, they become active responders, capable of both protective and damaging roles. By influencing neuronal health, regulating inflammatory responses, and responding to neurotransmitter signals, T cells and macrophages serve as pivotal players in the CNS’s response to injury, infection, and chronic disease.

Through complicated signaling networks, immune cells interact dynamically with glial cells, neurons, and the ECM, shaping the immune response to fit the CNS’s needs in both homeostatic and inflammatory contexts. These interactions allow immune cells to support neuronal health, modulate glial responses, and remodel the ECM during injury, demonstrating their pivotal roles in CNS maintenance and repair.

### 3.5. ECM

Alongside multifaceted cell–cell interactions, the CNS relies on an elaborate and highly specialized ECM. The extracellular matrix of the adult CNS is a complex structure comprising various proteins and glycans throughout the brain’s extracellular space, supporting cell survival, activity, and localization [6]. Acting as a source of molecular cues, the ECM also anchors neural cells in specific regions, stabilizing synapses, regulating synaptic plasticity, and preventing aberrant remodeling. In general, it serves as a structural and biochemical support matrix for CNS cells, providing physical scaffolding and containing molecules essential for cell signaling [79,97,98].

The ECM has three primary compartments in the adult CNS: the basement membrane, interstitial matrix, and perineuronal nets. The basement membrane, located between endothelial cells and neural tissue, maintains BBB integrity and supports angiogenesis. The interstitial matrix, spread throughout the CNS parenchyma, contributes to signal transmission and structural support, while the perineuronal net, surrounding the neuronal soma, protects neuronal health, stabilizes synaptic integrity, and supports memory processes. Together, these ECM structures play vital roles in CNS stability and plasticity [98,99,100,101].

Neural ECMs differ significantly from those in other tissues, which are primarily composed of fibrous proteins like collagen, fibronectin, and laminin. By contrast, the ECM of the CNS comprises a distinct and loosely structured network primarily composed of hyaluronan, sulfated proteoglycans, and tenascins. The perineuronal net is composed largely of hyalectans—a complex network of hyaluronans and lecticans, including aggrecan, brevican, neurocan, and versican [97]. Both neurons and glial cells contribute to the production and organization of neural ECMs, with ECM expression tightly regulated throughout CNS development [98,100,102].

Following initial damage to CNS tissues caused by traumatic injury or degenerative processes, inflammatory responses within the CNS actively remodel the neuronal ECM to limit the spread of neuronal damage and facilitate tissue recovery. These dynamic ECM alterations regulate gene expression related to ECM synthesis and also modify existing ECM components through post-translational mechanisms, such as proteolytic cleavage, fragmentation, or the release of glycosaminoglycan (GAG) residues from core proteins. Depending on the nature of these modifications, they can either promote neuronal repair or exacerbate inflammatory cycles, potentially leading to chronic inflammation within the CNS [97,103].

ECM remodeling is a crucial process in the recruitment of immune cells to sites of injury or inflammation in the CNS. When tissue damage occurs, the ECM undergoes dynamic changes, such as the degradation of existing matrix components and the deposition of new ones, to create a permissive environment for immune cell infiltration. Proteolytic enzymes, like matrix metalloproteinases (MMPs), cleave ECM proteins, facilitating the movement of immune cells like microglia, macrophages, and peripheral leukocytes towards the damaged area [6,104,105]. The modified ECM not only provides physical pathways for immune cell migration but also alters the local biochemical signals that influence immune cell activation. For example, fragments of ECM components can act as signals that either induce or suppress immune cell activity, thus fine-tuning the inflammatory response. Proper ECM remodeling is essential for effective immune cell recruitment, the resolution of inflammation, and tissue repair, while dysregulated remodeling can contribute to chronic inflammation and hinder recovery in neuroinflammatory conditions [6,79].

The composition of the ECM significantly influences neuroimmune cell behavior, shaping how these cells respond to injury, inflammation, and disease [99]. This allows for a dynamic environment that can rapidly change in response to cellular activity, promoting or inhibiting various immune cell behaviors. For example, hyaluronan facilitates cell migration and signaling, which are essential for the recruitment of immune cells such as microglia and infiltrating leukocytes to sites of injury or inflammation [102].

Sulfated proteoglycans, like chondroitin sulfate proteoglycans (CSPGs), are particularly important in modulating neuroimmune interactions [99,106]. These molecules interact with immune cells and can have both inhibitory and regulatory effects depending on the context. In normal conditions, CSPGs help maintain the integrity of the neural ECM and support cellular functions, but during injury or neurodegenerative diseases, their expression increases, often contributing to glial scarring and limiting neuronal regeneration. This modification of the ECM can delay the resolution of inflammation and hinder the ability of neuroimmune cells to promote tissue repair effectively [96,107]. Additionally, tenascins, which are involved in cell adhesion and migration, can influence immune cell activation. For instance, tenascin-C is upregulated during neuroinflammatory conditions and has been shown to modulate glial cell responses, enhancing the secretion of pro-inflammatory cytokines. This can exacerbate inflammation and contribute to chronic neuroinflammatory conditions, altering the local immune environment and affecting the recovery processes of the CNS [6,99]

ECM-derived molecules, including hyaluronic acid fragments and proteins like osteopontin and tenascin-C, can indeed function as damage-associated molecular patterns (DAMPs) or danger signals, triggering immune responses and promoting inflammation. When these ECM components are fragmented or altered, these molecules interact with immune cell receptors, including Toll-like receptors (TLRs) and CD44, initiating intracellular signaling cascades that regulate immune cell activation, the stimulation of pro-inflammatory pathways, and the modulation of cell survival [6,99,105]. This interaction between ECM-derived molecules and immune cell receptors is crucial for coordinating the immune response in the CNS, ensuring that inflammation is appropriately triggered and resolved during tissue repair. However, dysregulated signaling or prolonged activation of these pathways can lead to chronic inflammation and exacerbate tissue damage [96,105,108].

Thus, the unique composition of the CNS’s ECM plays a critical role in regulating neuroimmune cell behavior, influencing how these cells are recruited, activated, and how they contribute to both inflammation and repair. The dynamic remodeling of the ECM in response to injury or disease can either promote healing or, conversely, contribute to pathological inflammation, underlining the importance of ECM in determining the outcome of neuroinflammatory processes.

### 3.6. Cytokines

Cytokines, a diverse group of small proteins, play a dual role in the brain, contributing to both brain homeostasis and neuroinflammation. As signaling molecules, cytokines regulate various cellular processes within the brain, including neuronal function, synaptic plasticity, immune responses, and tissue repair [68,109]. The cytokine network, comprising cytokines, their receptors, and regulatory molecules, is distributed throughout the brain and other bodily systems, undergoing tight regulation across one’s lifespan. Operating within complicate cascade patterns, cytokines may act synergistically or antagonistically, enabling cross-talk between various cell types and translating environmental signals into cellular responses [2,86].

In the CNS, cytokines are produced by multiple cell types, each contributing to immune and neuroregulatory functions. Microglia, as the primary immune cells of the CNS, are major sources of cytokines, particularly during inflammation, releasing TNF-α, IL-1β, and IL-6. Astrocytes also produce cytokines, both pro- and anti-inflammatory, which help maintain homeostasis and respond to injury, such as IL-10 and TGF-β. Neurons can release certain cytokines, like IL-1β, under specific conditions, which influence synaptic plasticity and neurogenesis. Additionally, endothelial cells of the blood–brain barrier and infiltrating peripheral immune cells can produce cytokines, especially during inflammatory or immune responses [2,36,110].

Based on their inflammatory effects, cytokines are generally classified as pro-inflammatory (e.g., IL-1, IL-6, TNF) or anti-inflammatory (e.g., IL-4, IL-10, TGF-β), reflecting their respective roles in regulating immune responses [82]. They regulate immune responses within the brain, maintaining a balance between pro-inflammatory and anti-inflammatory signals. Cytokines can initiate and amplify neuroinflammatory responses by promoting the production of additional cytokines and chemokines, leading to a positive feedback loop that sustains inflammation. Chronic neuroinflammation can disrupt normal brain function and contribute to the pathogenesis of neurological disorders [111,112]. Anti-inflammatory cytokines such as interleukin-10 (IL-10) and transforming growth factor-beta (TGF-β) help resolve inflammation and prevent tissue damage [2,64].

In homeostatic states, cytokines such as IL-6, IL-10, TNF-α, and IFN-γ modulate synaptic transmission and neuronal excitability, contributing to normal brain function and homeostasis [113,114]. They influence neurotransmitter release, neurogenesis, synaptic plasticity, and neuronal survival under specific conditions and within a certain concentration range. Although typically pro-inflammatory, IL-6 at lower levels can support neuronal differentiation and synaptic plasticity. It has been linked to enhanced learning and memory when regulated within an optimal range. Known for its anti-inflammatory effects, IL-10 can create a neuroprotective environment and promote neurogenesis by limiting inflammatory damage and supporting neural cell survival.

At low physiological levels, pro-inflammatory TNF-α supports synaptic scaling and plasticity, whereas elevated levels may exert neurotoxic effects. TNF released by hippocampal microglia is essential for synaptic plasticity by regulating synaptic scaling, increasing synaptic strength, and supporting long-term potentiation (LTP), a mechanism vital for learning and memory formation [36,114,115,116,117]. When neuronal activity decreases, microglial TNF upregulates synaptic AMPA receptors, thereby enhancing synaptic strength and enabling homeostatic neuronal excitability adjustments [117,118].

Pro-inflammatory cytokine IFN-γ can influence neurogenesis in specific brain regions and, in low concentrations, has been associated with cognitive enhancements. It has been shown to promote neuronal differentiation and influence synaptic plasticity. Thus, cytokines may play a dual role, where balance is crucial; they support neurogenesis and plasticity in healthy contexts, while dysregulation can lead to neuroinflammation and neurodegeneration [111,119].

Thus, cytokines are essential signaling molecules, mediating complex interactions between diverse cell types. Acting as both communicators and modulators, cytokines play a crucial role in coordinating the responses of cells within the CNS to maintain homeostasis, support neurodevelopment, and respond to injury. This intercellular signaling network allows cytokines to influence a wide array of processes such as synaptic plasticity, neurogenesis, and immune responses. The cytokine network functions in a highly dynamic and reciprocal manner, with cells responding to cytokine signals by adjusting their own cytokine production, creating feedback loops that refine and regulate cellular behavior. Under inflammatory or pathological conditions, these regulatory loops may amplify immune responses, altering cytokine production and potentially leading to neurotoxicity. However, anti-inflammatory cytokines help counteract these effects by dampening excessive inflammation and promoting tissue repair. This finely tuned cytokine interplay is fundamental to CNS resilience, allowing for adaptive responses to a changing environment and enabling the CNS to regulate complex functions from cellular health to higher cognitive processes.

### 3.7. Neurotrophic Factors

Neurotrophic factors are essential signaling molecules that facilitate cellular communication across the nervous system, playing key roles in modulating neuronal growth, survival, and functional adaptation. Produced by neurons, glial cells, and even peripheral tissues, these molecules, such as insulin-like growth factor-1 (IGF-1), GDNF, and BDNF, act as modulators in a network of cellular interactions [2,67,120]. They support neural plasticity by fostering synapse formation, dendritic branching, and neurotransmitter release, thus shaping neuronal circuitry and facilitating learning, memory, and cognitive processes. Neurotrophic factors also help the CNS adapt to environmental and internal changes by responding to physiological demands, stress, or injury. Their distribution across various brain regions underscores their dynamic role in maintaining and adjusting neural networks for optimal function [121].

Neurotrophic factors, which are particularly abundant in brain regions integral for plasticity, play a pivotal role in facilitating axonal and dendritic growth and remodeling, making them key mediators of neuronal remodeling and connectivity. These factors are crucial for neurotransmitter production, synapse formation, and proper synaptic function [122,123].

There is evidence suggesting that enhanced brain inflammation, often observed with aging or following immune challenges, may impair the brain’s ability to supply BDNFs needed for memory-related plasticity at hippocampal synapses [121]. Numerous studies indicate that inflammation negatively impacts BDNF expression in the brain, with peripheral immune challenges shown to affect different BDNF transcripts, implying that inflammation may influence specific isoforms of this neurotrophin [122,123,124,125]. This supports the hypothesis that BDNF modulation may be one mechanism through which inflammation disrupts brain function [126].

Glial cell line-derived neurotrophic factor (GDNF) plays a crucial role in modulating neuroinflammation, providing both neuroprotective and anti-inflammatory effects within the CNS. GDNF is primarily produced by glial cells, such as astrocytes, and its expression can be upregulated in response to injury or inflammatory stimuli, protecting vulnerable neurons and promoting their survival. In neuroinflammatory conditions, GDNF acts by enhancing neuronal resilience to inflammatory insults, inhibiting apoptosis, and promoting repair mechanisms [127,128].

Furthermore, GDNF has been shown to attenuate the activation of microglia and reduce the production of pro-inflammatory cytokines like TNF-α and IL-1β, thus limiting the cascade of inflammation that can lead to neurodegeneration. Additionally, GDNF supports synaptic plasticity and promotes axonal regeneration, both critical processes for recovery in inflammatory CNS diseases. As such, GDNF is not only fundamental for neuronal health but also emerges as a key regulatory factor in maintaining the balance between neuroinflammatory responses and neuroprotection, making it a target of interest in therapies for neurodegenerative and neuroinflammatory disorders [120,127].

IGF-1 also plays a vital role as a neurotrophic factor, with deficiencies linked to cognitive impairment and dementia in older adults. Evidence suggests that synaptic function declines with age, and reduced IGF-1 levels contribute to diminished information processing in an aging brain [129]. Interestingly, recent findings propose an optimal IGF-1 level for cognitive functioning, as both high and low levels of IGF-1 have been associated with poorer cognition [130].

Thus, neurotrophic factors are indispensable within the CNS, acting as dynamic mediators that support cellular communication, adaptative plasticity, and resilience across a spectrum of physiological and pathological states. By modulating neuronal survival, synaptic integrity, and the complex interplay among glial cells and neurons, these molecules enable the nervous system to adapt to environmental changes and maintain cognitive function throughout aging. The regulatory role of neurotrophic factors is essential not only for fostering neurodevelopment and synaptic strength but also for safeguarding neural networks against degenerative processes and inflammation, highlighting their profound impact on both immediate and long-term CNS health.

### 3.8. Neurotransmitters

Neurotransmitters like glutamate and gamma-aminobutyric acid (GABA) play essential roles in maintaining homeostasis in the CNS but are also critical in neuroinflammatory responses [131]. In a homeostatic state, glutamate and GABA maintain a delicate balance between excitatory and inhibitory signaling, essential for processes like synaptic plasticity, learning, and memory. Glutamate, the primary excitatory neurotransmitter, is released from presynaptic neurons and binds to receptors such as NMDA and AMPA on postsynaptic neurons, facilitating excitatory signaling. GABA, on the other hand, is the primary inhibitory neurotransmitter and binds to GABA_A and GABA_B receptors, counterbalancing glutamate activity to prevent overexcitation and maintain neural stability [131,132].

During neuroinflammation, however, the dynamics of glutamate and GABA signaling can become disrupted. Activated immune cells, such as microglia and astrocytes, release glutamate as part of their response to injury, which can lead to elevated extracellular glutamate levels. This excess glutamate can overstimulate neurons, causing excitotoxicity, a process where the prolonged activation of NMDA and AMPA receptors leads to calcium overload in neurons, ultimately triggering cell damage or death. This excitotoxic environment can exacerbate inflammation, creating a cycle of neurodegeneration and immune activation [42,133,134].

GABA signaling is also affected in neuroinflammatory conditions. For instance, neuroinflammation can reduce GABAergic inhibition by decreasing GABA synthesis or impairing GABA receptor function, disrupting the inhibitory balance needed to counteract excitotoxicity [135,136]. Additionally, inflammatory cytokines released by microglia and astrocytes can interfere with GABA receptor expression and signaling, reducing the brain’s protective inhibitory responses. The imbalance between excitatory glutamate and inhibitory GABA signaling contributes to neuronal dysfunction and worsens neuroinflammation, making these neurotransmitters key players in the progression of CNS diseases [133,134,137].

Thus, glutamate and GABA are essential for maintaining a balanced and functional CNS; however, during neuroinflammation, their altered dynamics contribute to a cascade of harmful effects. Excess glutamate release drives excitotoxicity, while impaired GABA signaling weakens inhibitory control, together creating an environment where neuronal damage and immune activation reinforce each other. This shift from homeostasis to dysregulation not only accelerates neurodegeneration but also perpetuates chronic inflammation, worsening the impact on CNS health and complicating recovery processes.

## 4. The Dynamic Interactions Between Neuroimmune Cells and Their Mediators

After detailing the main key players involved in neuroimmune interactions in the previous section, we will now briefly unfold how these dynamic interactions between neuroimmune cells and their mediators can function under both homeostatic and neuroinflammatory conditions. As highlighted earlier, the CNS operates as a dynamic and complex network in which multiple cell types and molecular mediators work together to maintain tissue homeostasis and respond to challenges. Under homeostatic conditions, this network ensures proper neuronal function, regulates immune responses, and adapts to environmental stimuli. However, in neuroinflammatory conditions, the equilibrium of this network can be disrupted, leading to maladaptive responses that contribute to disease progression. In this interdependent and multilayered system, neuroimmune cells, including microglia, astrocytes, neurons, peripheral immune cells, and a variety of mediators, such as cytokines, neurotrophins, ECM components, and neurotransmitters, interact to regulate CNS function.

### 4.1. Homeostatic Conditions

**Figure 4:**
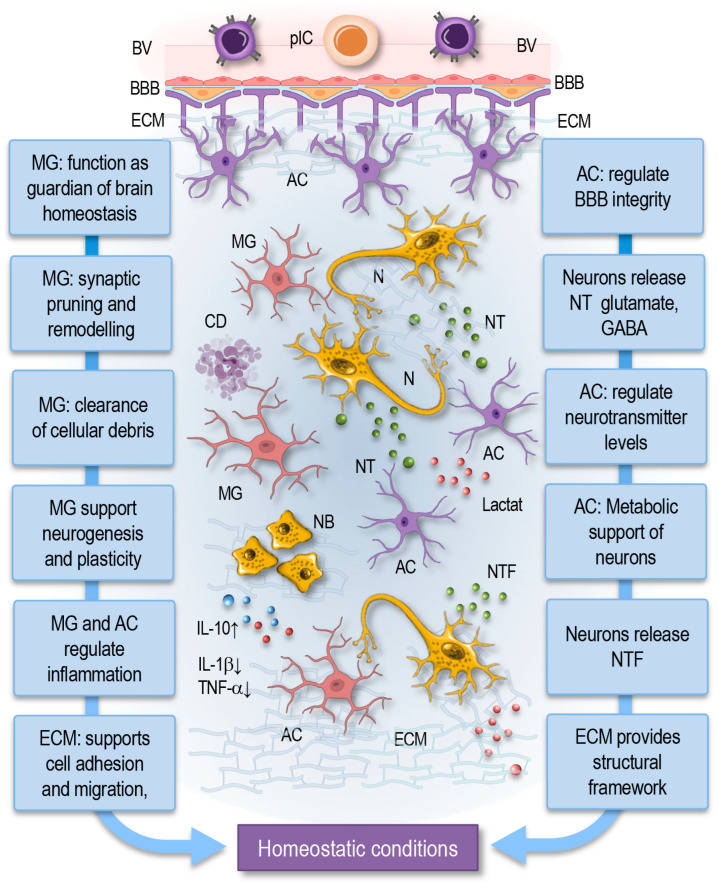
In a healthy CNS, a tightly regulated interplay among neurons, glial cells (microglia and astrocytes), and the extracellular matrix maintains functional homeostasis. Microglia monitor the environment, perform synaptic pruning, and clear cellular debris, while astrocytes regulate neurotransmitter levels, support neuronal metabolism, and reinforce the blood–brain barrier. Neurons communicate through neurotransmitters and release neurotrophins like BDNF to support synaptic stability. The ECM provides structural support and modulates growth factor availability. Low levels of cytokines facilitate intercellular communication, balancing immune activity without excessive inflammation, thus preserving CNS adaptability and resilience. BV: blood vessel; pIC: peripheral immune cells; BBB: blood–brain barrier; ECM: extracellular matrix; MG: microglia; AC: astrocytes; N: neuron; CD: cellular debris; NT: neurotransmitter; NTF: neurotrophins; NB: neuroblasts; IL: interleukin; ↑: increase; ↓: decrease; TNF: tumor necrosis factor.

In a healthy CNS, a tightly regulated balance of interactions among various cell types and signaling molecules forms the foundation of functional homeostasis (Figure 4). Each component contributes to the global system, which acts to preserve neuronal health, regulate immune activity, and respond dynamically to environmental stimuli [1]. Microglia, the resident immune cells of the brain, continuously monitor their environment through their highly motile processes. They play a critical role in synaptic pruning, removing excess or damaged synapses to ensure optimal neural network connectivity. Microglia are also involved in the phagocytosis of cellular debris, thus maintaining the integrity of the CNS [2,12,28,38,113].

Astrocytes, another major glial cell type, maintain synaptic homeostasis by regulating neurotransmitter levels, specifically by clearing excess glutamate from synaptic clefts to prevent excitotoxicity. They also support neurons metabolically by supplying lactate as an energy substrate. Additionally, astrocytes form a key component of the blood–brain barrier (BBB), providing physical and chemical support to prevent harmful substances from entering the CNS. By extending their end-feet processes around blood vessels, astrocytes contribute to the maintenance of tight junctions between endothelial cells, which are crucial for the BBB’s integrity (Figure 4). They also secrete various signaling molecules, such as cytokines, growth factors, and extracellular matrix components, which regulate the permeability and stability of the blood–brain barrier [11,61,74].

The BBB plays a crucial role in maintaining central nervous system homeostasis by regulating the exchange of substances between the bloodstream and the brain. It restricts the entry of potentially harmful molecules, pathogens, and immune cells while allowing the selective transport of essential nutrients and waste products. The tight junctions between endothelial cells, along with astrocytic end-feet and pericytes, form a dynamic barrier that supports a stable neural environment necessary for proper synaptic function and neural signaling [138].

Neurons communicate with one another and with glial cells through the release of neurotransmitters such as glutamate, GABA, and acetylcholine. These signaling molecules ensure the proper balance of excitatory and inhibitory signals required for synaptic plasticity, learning, and memory. Neurons also release neurotrophins, such as BDNF, which promote survival, growth, and the differentiation of neurons, thus contributing to synaptic stability and plasticity [9,73,127].

The extracellular matrix serves as a scaffold within the CNS. It provides a structural framework for neural and glial cells while also regulating the availability of growth factors and signaling molecules that modulate neural function [96,99,101,107].

In this homeostatic state, cytokines, typically associated with immune responses, are present at low levels and perform vital roles in intercellular communication. Pro-inflammatory cytokines, such as TNF-α and IL-1β, are involved in synaptic scaling, ensuring neuronal circuits remain adaptable. Anti-inflammatory cytokines like IL-10 help maintain immune surveillance without provoking excessive inflammatory responses [36,38,66,68,86,117].

The coordinated interplay between microglia, astrocytes, neurons, and the ECM ensures that the CNS remains adaptable, resilient, and capable of responding to environmental changes or mild insults without triggering an inflammatory cascade.

### 4.2. Neuroinflammatory Conditions

**Figure 5:**
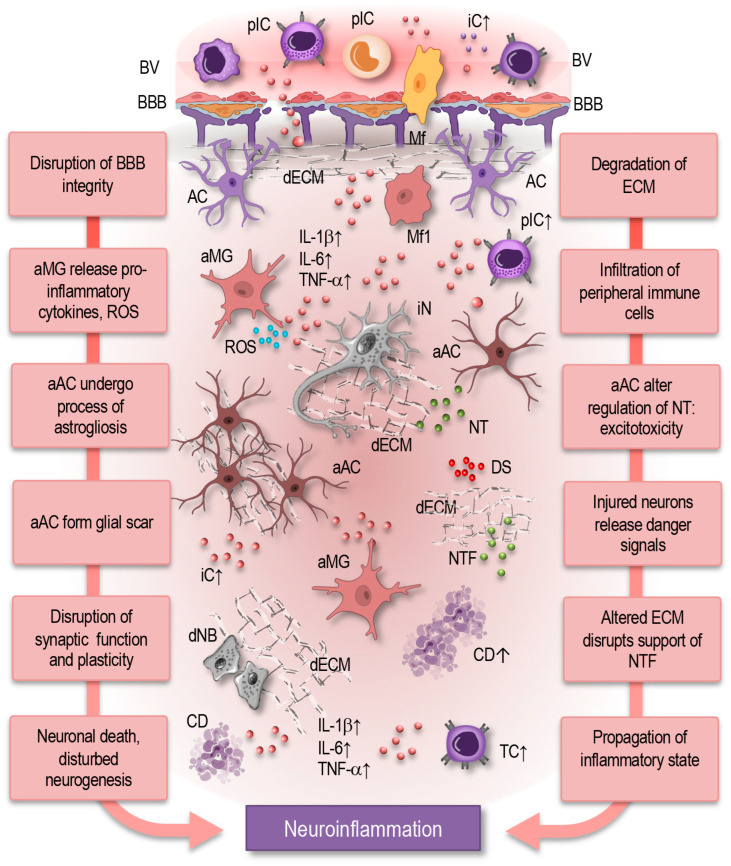
In pathological conditions such as trauma, infection, or neurodegenerative disease, the CNS’s balanced network becomes dysregulated, leading to neuroinflammation. Microglia shift to an activated state, adopting a neurotoxic profile and releasing pro-inflammatory cytokines such as ROS and nitric oxide that exacerbate neuronal injury. Astrocytes undergo reactive astrogliosis, forming glial scars that can limit damage spread but hinder regeneration by releasing pro-inflammatory mediators that sustain inflammation. Injured neurons release danger signals, activating surrounding glia and attracting peripheral immune cells, further intensifying inflammation. Elevated cytokine levels disrupt synaptic function and neurotrophin signaling, weakening neuronal resilience. The ECM is degraded, compromising structural integrity and enabling peripheral immune cell infiltration, which perpetuates the inflammatory state. BV: blood vessel; pIC: peripheral immune cells; ↑: increase; ↓: decrease; BBB: blood–brain barrier; Mf: macrophages; Mf1: inflammatory macrophages; AC: astrocyte; dECM: depredated extracellular matrix; aMG: activated microglia; ROS: reactive oxygen species; aAC: activated astrocytes; IL: interleukin; TNF: tumor necrosis factor; iN: injured neuron; DS: danger signal; CD: cellular debris; NT: neurotransmitter; NTF: neurotrophins; dNB: disturbed neuroblasts; TC: T cells.

In pathological states, such as trauma, infection, or neurodegenerative diseases, this finely tuned network becomes dysregulated, leading to neuroinflammation (Figure 5). During neuroinflammation, the BBB’s integrity is often compromised, leading to increased permeability. This disruption allows the infiltration of peripheral immune cells and inflammatory mediators into the CNS, amplifying local inflammation [139]. In these conditions, neuroimmune cells and mediators adopt altered phenotypes and initiate responses that can either be protective or detrimental, depending on the nature and duration of the insult.

Microglia, upon sensing damage or infection, change from a surveillant state to an activate state. Activated microglia can adopt a neuroprotective phenotype by clearing debris, releasing anti-inflammatory cytokines, and promoting repair. However, persistent activation leads to a neurotoxic phenotype, characterized by the secretion of pro-inflammatory cytokines such as TNF-α, IL-1β, and IL-6, as well as reactive oxygen species (ROS) and nitric oxide (NO), all of which contribute to neuronal injury and exacerbate the inflammatory response [1,5,8,28,32,38,39,42,61,73,79,88].

Astrocytes also undergo a dramatic shift in function during neuroinflammation, a process termed astrogliosis. Reactive astrocytes upregulate the production of pro-inflammatory mediators and form a glial scar around the injured tissue. While this scar can serve as a protective barrier to prevent the spread of damage, it can also inhibit neural regeneration and contribute to a chronic inflammatory environment. Reactive astrocytes may further amplify inflammation by releasing cytokines, such as IL-6 and TNF-α, and by altering their regulation of neurotransmitters like glutamate, potentially leading to excitotoxicity [4,10,51,52,53,59,61,62,64,68,74,80].

While primarily targets of neuroinflammation, neurons can also contribute to the inflammatory response. Injured neurons can release alarmins, such as ATP and high-mobility group box 1 (HMGB1), which act as danger signals to activate nearby microglia and astrocytes. This amplifies the inflammatory cascade, further attracting peripheral immune cells like T lymphocytes and macrophages. The recruitment of peripheral immune cells to the CNS, once a tightly regulated process, becomes dysregulated, exacerbating the inflammatory response and potentially leading to autoimmunity, as seen in multiple sclerosis [9,89,110].

Cytokines take on a central role in neuroinflammation, with elevated levels of pro-inflammatory mediators such as TNF-α, IL-1β, and IL-6 driving the inflammatory response. These cytokines not only activate microglia and astrocytes but also disrupt synaptic function and plasticity. They can impair neurotrophin signaling, particularly BDNF, leading to neuronal dysfunction and increased vulnerability to injury [2,36,37,66,89].

The ECM also undergoes significant changes during neuroinflammation. Matrix metalloproteinases, enzymes involved in the degradation of ECM components, are upregulated, leading to the breakdown of the structural integrity of the ECM. This process can facilitate the infiltration of peripheral immune cells into the CNS, further amplifying the inflammatory response. The altered ECM also impairs the availability of growth factors and signaling molecules, thereby disrupting the support that neurons and glial cells require for repair and recovery [6,97,99,105]. 

In summary, under homeostatic conditions, neuroimmune cells and mediators form a multilayered network that maintains CNS function and resilience. However, in neuroinflammation, this network becomes dysregulated, with altered phenotypes and signaling pathways leading to a cascade of inflammatory events that can be either protective or harmful. The transition from homeostasis to neuroinflammation represents a shift in the balance of this complex network, with long-term consequences for neural function and disease progression. In the following section, we aim to understand these intricate interactions through the lens of a multilayer network approach.

## 5. Understanding Complex Networks in Neuroimmune Interactions Through Multilayer Network Models

Complex systems, including cellular networks, brain activity, and social networks, consist of intricate, interdependent connections that can be effectively captured through network models. Network graphs are commonly used to represent these connections, with nodes symbolizing entities and edges representing the relationships between them [140,141,142].

In our previous work, single-layer network models were applied to examine interactions among circulating pro- and anti-inflammatory biomarkers, hormones, neurotrophic and metabolic factors, immune cells, and cognitive performance measures. This approach revealed significant variations in network topology influenced by factors such as sex and cytomegalovirus serostatus [143]. However, real-world systems often interact across multiple domains, creating complex interdependencies that require a more nuanced, multilayer network approach.

In the context of the CNS, understanding the multifaceted interplay of cellular and molecular actors can greatly benefit from the MLN framework. This model provides a holistic view of how each interaction layer contributes to system functionality and resilience [144,145,146,147]. MLNs reveal unique structural features and evolving behaviors, such as enhanced diffusion, mesoscale organization, and phase transitions between healthy and inflammatory states, that may be overlooked in isolated layers [144,146]. For instance, in human interactomes, these adaptable, multilayer structures help us understand how cellular and neural communication pathways function under both normal and stress conditions, highlighting the adaptive properties of key hubs with multiple interconnections.

A network-based approach, particularly using MLN models, is crucial for analyzing complex systems. First, systems represented as networks or graphs can be assessed based on their topology, offering a quantitative description of interactions and dynamic behaviors. Key graph-theoretical metrics, such as clustering coefficient, characteristic path length, and local/global efficiencies, provide insights into how a system balances segregation and integration [141,148,149,150]. Centrality measures, like node degree, closeness, betweenness, eigenvector, and information centrality, further reveal the structural and functional importance of individual nodes within the network [144,149].

MLN approaches reveal that genes linked to the same disease cluster together in specific “disease modules” within molecular networks, rather than being randomly dispersed [151,152,153]. According to the so-called “disease module hypothesis”, cellular components related to a particular disease localize within specific neighborhoods of the human interactome—a map of biologically significant molecular interactions [152]. For example, the asthma module overlaps with immune response mechanisms shared across immune-related diseases [153].

Even when applying the modularity analysis to single-layer networks, it has been shown that the networks under consideration (multiple interactions among various inflammatory and cognitive biomarkers in groups with different sexes and CMV serostatuses) exhibited in all cases highly differentiated and heterogeneous modular organization (with high modularity values), where cognitive performance nodes shared the same module as inflammatory biomarkers, indicating strong interdependences between these entities [143]. Thus, network modularity and its underlying modular structure, especially in MLNs, indicate future important issues of network organization and functioning.

In immune system networks, for example, nodes represent cells, while links denote communication through signaling molecules like cell surface receptors or secreted factors. [154]. Mapping these networks is achievable through mass spectrometry, which allows for the comparison of proteomes in immune cell populations by measuring intracellular and secreted proteins across different stimuli. One study identified over 180,000 high-confidence interactions between 460 receptors and 300 ligands using this approach [155]. Network analysis has illuminated core principles of intracellular communication, revealing distinct communication patterns among immune cells following their activation. The complicated interactions among immune cells bear a strong resemblance to a social network [154]. To respond effectively to diverse external and internal threats, immune cells must work in a highly cooperative and coordinated manner, with communication playing a crucial role in this process. The large-scale proteomics analyses conducted by Rieckmann et al. [155] offer valuable insights into the fundamental architecture of these complex communication networks among different immune cell types, which have proven useful in predicting disease associations with cytokines [154,155].

**Figure 6:**
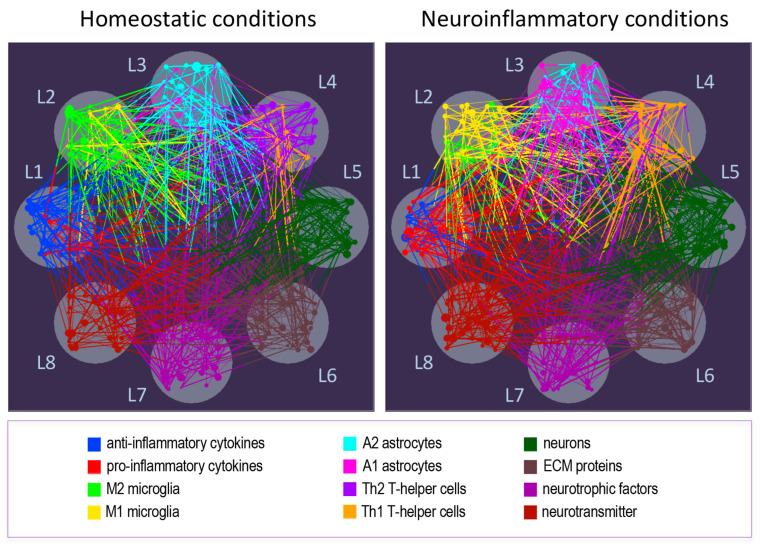
Each MLN consists of eight layers (L1–L8) with about 30 nodes in each layer. Nodes in different layers and corresponding edges are indicated by color. To illustrate the dynamic interplay in homeostatic (left) and neuroinflammatory (right) conditions or states, the first four layers (L1–L4) are split into clusters representing pro- and anti-inflammatory mechanisms. These clusters vary in node count and connectivity based on the inflammatory state; in the homeostatic state, anti-inflammatory cytokines (blue) predominate, whereas in the neuroinflammatory state, pro-inflammatory cytokines (red) become more prominent. Similar patterns apply to other cell types in layers L2 to L4. Microglia in layer 2 shift between M2 (green) and M1 (yellow) states; astrocytes in layer 3 vary between A2 (cyan) and A1 (pink) subtypes, and immune cells in layer 4 separate into Th2 (violet) and Th1 (sandy brown) T-helper subtypes, with state changes corresponding to homeostatic and neuroinflammatory conditions, respectively. For simplicity, neuronal, ECM, neurotrophic, and neurotransmitter layers (L5–L8) were not distinguished by pro- or anti-inflammatory impacts in the model; however, these layers remain dynamic, receiving inputs from the inflammatory layers and influencing the system’s behavior as described in the text. L1: cytokines; L2: microglia; L3: astrocytes; L4: immune cells; L5: neurons; L6: ECM; L7: neurotrophic factors (NTFs); L8: neurotransmitters.

In Figure 6, we present a simplified representation of the interdependent interactions among the eight key players considered in the previous sections. Constructed using Monte Carlo simulations, this MLN model reflects insights into the complex dynamics of neuroimmune cell signaling in both homeostatic and neuroinflammatory conditions. Through this schematic model, we may observe how these eight interconnected layers form an MLN that supports adaptability, resilience, and diverse response patterns. This multilayered perspective elucidates how individual components interact to propagate signals, regulate inflammation, and maintain CNS structural integrity, offering a refined lens on network organization and dynamics.

When applied to MLNs, such measures may capture interactions both within and between network layers, providing a comprehensive view of the system’s multidimensional connectivity [144,145,146,149]. A vital property of networks, especially those in biological and physical systems, is their robustness—the capacity to sustain functionality despite node or edge disruptions [156,157,158]. In complex real-world networks, the targeted removal of high-ranking nodes or links leads to the most significant damage, whereas networks with randomly distributed link weights are more vulnerable to disruptions [159,160]. Scale-free networks, typical in biological systems, are resilient to random node failures but are sensitive to the removal of high-degree nodes, which can fragment the network or limit communication between distant nodes [161].

Moreover, MLNs present distinct resilience dynamics compared to single-layer networks. Interdependencies between layers mean that failures in one layer can propagate through others, increasing fragility [162,163]. Interestingly, network topology plays a significant role in resilience; interdependent scale-free networks are more susceptible to targeted attacks compared to Poisson networks with similar average degrees. In multiplex networks, where each node replicates across various layers, stress impacts the entire system uniformly, whereas in systems with varying interdependencies, layers with a greater number of connections (superdegree) are more vulnerable [144]. This reveals that network resilience is shaped by both inter-layer dependencies and internal structure, highlighting new dimensions of robustness in complex, interdependent systems.

For example, the CNS’s resilience to inflammatory insults relies on the robustness of its cellular and molecular interactions. An MLN can demonstrate how certain nodes, such as high-impact signaling molecules or central glial cells, may act as crucial hubs. Their disruption can propagate through multiple layers, leading to widespread effects like sustained inflammation or neurodegeneration. This highlights the need to identify critical hubs within each layer, as targeting them could mitigate adverse outcomes associated with neuroinflammation.

In summary, linking the multilayer network approach to neuroinflammation allows us to understand the complex interplay among cellular, molecular, and signaling components in the CNS during immune responses. Neuroinflammation is not solely a localized response but a highly networked process, involving interactions across multiple layers of cellular and molecular networks that mediate inflammation, neuroprotection, and repair. An MLN model provides a powerful tool to capture the cross-layer dependencies and cascade effects that can occur during inflammatory responses in the CNS. Furthermore, analyzing neuroinflammation through an MLN enables us to see how targeted therapies (e.g., blocking specific cytokines) in one layer may influence other layers, such as neuronal function or metabolic stability. This approach may reveal unanticipated therapeutic impacts, as well as potential cascade effects, helping to refine strategies aimed at reducing neuroinflammation without triggering unwanted side effects.

## 6. Conclusions

Understanding the nervous system as a dynamic system with interdependent cellular and molecular networks sheds light on the mechanisms that underpin both its adaptability and vulnerability to neuroinflammation. By characterizing key players like microglia, astrocytes, neurons, immune cells, and essential signaling molecules, we gain insights into how these elements maintain homeostasis and support neuroimmune interactions that are essential for CNS resilience. However, under neuroinflammatory conditions, these interactions can become dysregulated, leading to potential pathologies. Approaching these networks from a multilayered perspective offers a more integrative view of the complex neuroimmune landscape, highlighting potential intervention points to restore balance and support CNS health. This article underscores the importance of exploring neuroimmune communication not only to understand disease mechanisms but also to guide future therapeutic strategies in managing neuroinflammatory disorders.

In general, advances in network biology, informed by complex network theory, reveal universal principles that apply across biological, physical, social, and technological domains. The emerging insights reveal that cellular function is less about individual molecules and more about the structured, quantifiable patterns of interactions among them. By uncovering these patterns, network theory helps to elucidate cellular behaviors and responses, driving new approaches in experimental biology. By studying multilayer networks, scientists are uncovering the fundamental rules that govern complex systems, advancing our understanding of everything from cellular dynamics to social and ecological systems and laying the groundwork for new, integrative approaches to biology and medicine.

## Data Availability

No new data were created or analyzed in this study.
